# A brief online transdiagnostic measure: Psychometric properties of the Overall Anxiety Severity and Impairment Scale (OASIS) among Spanish patients with emotional disorders

**DOI:** 10.1371/journal.pone.0206516

**Published:** 2018-11-01

**Authors:** Alberto González-Robles, Adriana Mira, Clara Miguel, Guadalupe Molinari, Amanda Díaz-García, Azucena García-Palacios, Juana M. Bretón-López, Soledad Quero, Rosa M. Baños, Cristina Botella

**Affiliations:** 1 Department of Basic and Clinical Psychology, and Psychobiology, Universitat Jaume I, Castellon, Spain; 2 CIBER Fisiopatología Obesidad y Nutrición (CIBERObn), Instituto Salud Carlos III, Madrid, Spain; 3 Department of Personality, Evaluation and Psychological Treatments, Universidad de Valencia, Valencia, Spain; Universita degli Studi Europea di Roma, ITALY

## Abstract

The Overall Anxiety Severity and Impairment Scale (OASIS) is a self-report questionnaire designed to evaluate the severity and functional impairment associated with anxiety. Given its transdiagnostic nature, it can be used indistinctly across anxiety and depressive disorders. In this study, the psychometric properties of the online version of the OASIS were evaluated in a Spanish clinical sample with emotional disorders. Patients (n = 583) with anxiety (n = 250) and depression (n = 333) with a mean age of 37.21 (SD = 12.22), underwent a diagnostic interview and questionnaires assessing anxiety, depression, positive and negative affect, and quality of life. Factorial structure, internal consistency, convergent and discriminant validity, cutoff scores, and sensitivity to change were analyzed. Confirmatory Factor Analysis yielded a unidimensional factor structure, consistent with previous validations of the instrument. The analyses showed good internal consistency and adequate convergent and discriminant validity, as well as sensitivity to change. A cutoff score of 7.5 was found to meet the criteria used in this study to select the optimal cutoff point. Overall, in this study, the psychometric properties of the online version of the OASIS were found to be appropriate. The brevity and ease of use of the OASIS support its adequacy as a valid measure of anxiety severity and impairment in Spanish clinical samples with anxiety and depression.

## Introduction

Anxiety and depressive disorders, also known as emotional disorders (ED), are prevalent [1,2] and costly [3,4] and an important cause of suffering and disability worldwide [5,6]. Moreover, the literature has shown the high comorbidity rates among anxiety disorders, and between anxiety and depressive disorders [7].

Along with depression, anxiety disorders are one the most prevalent disorders, with a 12-month prevalence of 18.1% [8], and a lifetime prevalence of 28.8% [1]. In Spain, the 12-month prevalence of an anxiety disorder has been estimated at 6.2%, and the lifetime prevalence at 9.3% [9]. Anxiety disorders are associated with important impairments [10], significantly poorer quality of life [11], and high rates of comorbidity with other anxiety disorders and with depression [2,7]. Therefore, the development of treatments for anxiety is a key aspect in addressing this important health problem. Moreover, the impact of these interventions cannot be ascertained without the use of appropriate assessment instruments. In this vein, despite the importance of evidence-based assessment (i.e. the use of research and theory to guide the selection of the most appropriate instrument for the assessment of a specific construct) [12], the attention paid to assessment is more recent than the importance given to evidence-based treatments, first described in a book published ten years earlier [13]. Therefore, the development and validation of rigorous assessment tools is an important task for researchers and clinicians involved in the assessment and treatment of anxiety disorders. In this vein, the need for digital versions of pen and paper scales has increased exponentially due to the proliferation of web-based interventions [14,15]. Nevertheless, the literature highlights that paper and online versions of the same instrument show strong correlations but may differ in psychometric properties [14]. Therefore, as research on web-based treatments advances, it becomes crucial to develop and validate assessment instruments that can be applied online [16].

Currently, there are a number of measurement tools to assess overall anxiety, such as the Beck Anxiety Inventory [17] or the State-Trait Anxiety Inventory [18]. These scales have been translated into Spanish and validated in previous research [19–21]. Additionally, several instruments have been developed and validated for the assessment of the symptoms associated with each of the different anxiety disorders (i.e. disorder-specific symptoms), such as the Penn State Worry Questionnaire [22] for generalized anxiety disorder, the Panic Disorder Severity Scale [23] for panic disorder and/or agoraphobia, and the Social Interaction Anxiety Scale [24] for social anxiety disorder. However, all these instruments focus on the assessment of individual anxiety symptoms (i.e. the occurrence of cognitive, emotional, and physiological symptoms), but they do not provide a measure of the global severity and impairment associated with these problems.

The Overall Anxiety Severity and Impairment Scale (OASIS) is a short scale made up of 5 items developed to assess the severity and impairment associated with anxiety disorders and/or symptoms [25–27]. Two advantages of the OASIS include its brevity and ease of use and its transdiagnostic nature. Regarding brevity, the need for short scales (i.e. less than 10 items) has been highlighted in the literature [25]. Several advantages have been indicated in this regard, such as the fact that it is an easier way to obtain relevant data in clinical settings such as primary care (where resources are normally limited) [25,28] or that symptoms can be monitored throughout a treatment [12]. For instance, this latter aspect might be particularly useful when it is necessary to evaluate anxiety symptoms repeatedly throughout a treatment (i.e. after each treatment module or session). Finally, in a more general way, even though brevity might compromise a scale’s validity [29], compared to longer scales, the use of shorter scales provides a more efficient way to collect data and maximize the representativeness of the sample [28]. In addition, from a transdiagnostic perspective, it is logical to develop and validate measures that capture the severity and impairment of anxiety disorders, regardless of the specific anxiety disorder suffered by the patients [25,30]. Following the DSM-IV-TR guidelines to establish the severity and associated impairment caused by anxiety, the five items on the OASIS were developed in an attempt to capture the most important domains of anxiety that are common to all anxiety disorders, namely, severity (i.e. frequency and intensity), behavioral avoidance, and functional impairment (i.e. work and social interference) [26]. Because the OASIS focuses on the severity and functional consequences of anxiety, rather than the occurrence of specific anxiety symptoms (which might vary depending on the specific presentation of each patient), the scale can be used in a transdiagnostic manner across different anxiety disorders. Given the theoretical and empirical association between anxiety and depression [2] and the high comorbidity rates between these disorders, the scale can also be used to assess the severity and impairment of anxiety in individuals with depression.

Previous versions of the OASIS have been validated in both clinical [25,31–33] and non-clinical samples [26,27]. In sum, the OASIS has shown sound psychometric properties in the existing literature. Nevertheless, to our knowledge, the OASIS has not yet been validated in Spanish clinical samples with anxiety and depressive disorders. Furthermore, most of the previous work in clinical populations has focused on patients with principal diagnoses of anxiety disorders, with some exceptions [31,32] that also included patients with a principal diagnosis of depression. However, in these studies, the proportion of patients with depression was low, compared to patients with anxiety [31]. Regarding the online validation of the OASIS, to our knowledge, only one study in the literature has used online surveys [32]. However, even though this study showed good psychometric properties, it relied on patients’ self-reports to establish a formal diagnosis, rather than well-validated measures such as diagnostic interviews or self-report questionnaires.

### Current study

In this study, we aimed to contribute to filling this gap by analyzing the psychometric properties of the OASIS in two clinical subsamples of individuals with emotional disorders: a subsample with a principal diagnosis of anxiety (n = 250) and a subsample with a principal diagnosis of depression (n = 333). Specific objectives were: a) to examine how the scale performs in patients with anxiety disorders vs. depressive disorders; b) to examine the scale’s factorial structure, reliability, and validity; c) to obtain cutoff scores; and d) to analyze sensitivity to change. To the best of our knowledge, this is the first study to evaluate the psychometric properties of the online version of the OASIS in a sample of adults with anxiety and depressive disorders in the Spanish population.

## Methods

### Spanish translation of the OASIS

First, a native Spanish-speaker who was aware of the purpose of the study translated the OASIS items from English to Spanish. Second, a Spanish-English bilingual speaker who was not familiar with the questionnaire performed a back-translation from Spanish to English. The person involved in the translation process is a native English speaker who has been living in Spain for many years and is fluent in both languages. The two English versions were compared, and the Spanish version of the OASIS was judged to be an accurate translation of the original English version.

### Procedure

The sample was recruited from patients attending the Emotional Disorders Clinic at Jaume I University (Castellon, Spain), whose principal focus is the treatment of ED using Information and Communication Technologies such as web-based interventions. Individuals who were waiting to receive an online treatment were invited to participate in the study, and those who agreed to participate provided written, informed consent. Only participants with a principal diagnosis of an emotional disorder (i.e. anxiety and depressive disorders) were considered for the study. All participants were assessed with a structured diagnostic interview, and a battery of questionnaires. All these measurement tools are described in detail in the Instruments section. The study was approved by the Ethics Committee of Universitat Jaume I.

### Participants

A total of 583 individuals with a mean age of 37.21 years (SD = 12.22; range: 18–68 years old) took part in the study. Most participants were female (n = 421; 72.21%), married or living with a partner (n = 273; 46.83%), and had completed higher education studies (n = 371; 56.3%). All of the participants were Caucasian. Regarding their diagnoses, 333 patients had a principal diagnosis of a mood disorder (i.e. major depressive disorder, dysthymic disorder, mood disorder not otherwise specified), and 250 had a principal diagnosis of an anxiety disorder. In all, more than half the sample had at least one comorbid anxiety or depressive disorder (53.5%). Diagnostic assessments were performed by pre-doctoral students who had been previously trained in the use of the diagnostic interview. A full description of the patients’ sociodemographic and clinical data is displayed in Table 1.

**Table 1:** Sociodemographic and clinical characteristics of the sample (n = 583).

Age in years, Mean (SD)	37.21 (12.22)	
**Sex, n (%)**	Female	421 (72.21)
	Male	162 (27.79)
**Relationship status, n (%)**	Single	235 (40.31)
	Married/de facto	279 (47.86)
	Divorced	62 (10.63)
	Widowed	7 (1.20)
**Education level, n (%)**	Basic	94 (16.12)
	Medium	179 (30.70)
	Superior	310 (53.17)
**Principal diagnosis, n (%)**	Major depressive disorder	318 (34.5)
	Generalized anxiety disorder	99 (17)
	Social anxiety disorder	57 (9.8)
	Panic disorder/agoraphobia	50 (8.6)
	Obsessive-compulsive disorder	14 (2.4)
	Dysthymic disorder	13 (2.2)
	Anxiety disorder NOS	12 (2.1)
	Specific phobia	10 (1.7)
	Postraumatic stress disorder	4 (0.7)
	Mood disorder NOS	2 (0.3)
	Intermittent explosive disorder	2 (0.3)
	Somatoform disorder	1 (0.2)
	Hypochondriasis	1 (0.2)
**Number of comorbid disorders, n (%)**	0	271 (46.5)
	1	210 (36)
	2	80 (13.7)
	≥ 3	22 (3.8)
**Symptom severity, Mean (SD)**	OASIS	8.69 (4.21)
	BAI	20.12 (11.80)
	BDI-II	23.39 (11.09)
	ODSIS	7.70 (4.91)
	PANAS-P	21.31 (7.49)
	PANAS-N	26.16 (7.95)
	QLI	4.79 (1.68)

OASIS = Overall Anxiety Severity and Impairment Scale; BAI = Beck Anxiety Inventory; BDI-II = Beck Depression Inventory-II; ODSIS = Overall Depression Severity and Impairment Scale; PANAS-P = Positive and Negative Affect Schedule-Positive Affect; PANAS-N = Positive and Negative Affect Schedule-Negative Affect; QLI = Multidimensional Quality of Life Questionnaire

### Instruments

#### Diagnostic interview

**Mini-International Neuropsychiatric Interview** [34]. The MINI is a short, structured clinical interview designed to perform diagnoses according to the DSM-IV and ICD-10 criteria. It has shown excellent test-retest and interrater reliability, as well as high predictive validity rates. The Spanish validation was used in this study [35].

#### Self-reported questionnaires

**Overall Anxiety Severity and Impairment Scale** (OASIS) [25]. The OASIS is a 5-item self-report scale that evaluates the frequency and severity of anxiety symptoms, the functional impairment related to these symptoms (i.e. school, work, home, or social impairment), and behavioral avoidance. Each item instructs respondents to endorse one of five responses that best describes their experiences over the past week. Response items are coded from 0 to 4, added together to obtain a total score ranging from 0 to 20. Previous studies have shown high internal consistency (α = 0.80), test-retest reliability, and convergent and discriminant validity [25–27]. In the current study, Cronbach’s alpha coefficient for the five items on the OASIS was good (0.86).

**Beck Anxiety Inventory** (BAI) [17]. This is a 21-item self-report questionnaire for the measurement of anxiety symptoms experienced during the past week. Each item is rated from 0 to 3 (i.e. not at all, mildly, moderately, severely), added together to obtain a maximum score of 63. The BAI has demonstrated good to excellent internal consistency in prior validations of the scale (.85-.94), as well as adequate convergent and divergent validity [20]. Cronbach’s alpha for the BAI in the present study was excellent (.91).

**Beck Depression Inventory** (BDI-II) [36]. The BDI-II is a 21-item self-report scale designed to assess depressive symptoms experienced during the past week. Items are rated on a Likert scale rated from 0 to 3, and the total score ranges from 0 to 63. The BDI-II has shown optimal validity and reliability in both clinical and nonclinical samples [36–38]. Cronbach’s alpha for the BDI-II in the present study was excellent (0.91).

**Overall Depression Severity and Impairment Scale** (ODSIS) [39]. The ODSIS consists of five items that measure the severity and impairment related to depression, as well as its interference with school, work, and social life. The measure has shown excellent internal consistency (α = 0.94 in an outpatient sample, 0.92 in a community sample, and 0.91 in a student sample) [39] and good convergent and discriminant validity. In the present study, the ODSIS showed excellent internal consistency (0.93).

**Positive and Negative Affect Schedule (PANAS)** [40]. The PANAS is a self-report measure that evaluates two dimensions on two independent scales: positive (PANAS-P) and negative affect (PANAS-N). Each scale is composed of 10 items coded in a range from 10 to 50 points. The PANAS has shown excellent convergent and divergent validity, as well as high internal consistency [40–42]. In the current study, Cronbach’s alpha was excellent for the PANAS-P (0.93) and good for the PANAS-N (0.88).

**Multidimensional Quality of Life Questionnaire** (QLI) [43]. The QLI is a self-report questionnaire that consists of 10 items aimed at assessing quality of life in ten areas: psychological well-being, physical well-being, emotional and social support, interpersonal functioning, self-care and independent functioning, community and service support, occupational functioning, self-realization, spiritual satisfaction, and an overall assessment of quality of life. The Spanish version of the QLI has shown good internal consistency and test-retest reliability in previous studies [44]. Cronbach’s alpha for the QLI in the present study was excellent (0.90).

### Data analysis

First, descriptive statistics (mean, standard deviation, skewness, and kurtosis) for the anxiety and depression subsamples were calculated for all the measures. Next, one-way ANOVAs were calculated to analyze whether there were significant differences in the scores on the OASIS based on gender, marital status, studies, and diagnosis. Furthermore, correlations between age and the OASIS score were calculated in order to study whether there were any associations between these variables. In addition, reliability was analyzed by calculating internal consistency indexes (Cronbach’s alpha) for the five items on the OASIS.

To analyze the factor structure of the OASIS, we performed Confirmatory Factor Analysis (CFA), a procedure based on Classical Test Theory (CTT) [45]. CFA models were estimated with maximum likelihood and robust corrections (MLR), given the scale’s non-normality and five-point response scale. Full Information Maximum Likelihood was employed to handle missing data. Following Norman et al. [27], a single latent factor with correlated error variances between items 1 and 2 was tested as the basis for the CFA model. Model fit was evaluated using several criteria, specifically, the chi-square test (*χ*^*2*^), comparative fitness index (CFI), Tucker-Lewis index (TLI), standardized root mean residuals (SRMR), and root mean square error of approximation (RMSEA). The following cutoff scores were used to determine good fit: CFI and TLI above .90 (better if above .95) and RMSEA below .08 [46]. Following recommendations by McNeish, An, & Hancock [47], factor loadings with their corresponding *p* values and the correlations between the error variances of the items were reported to evaluate the validity of the factor model. A correlation between the error variance of items 1 and 2 was expected because a response of 0 to item 1 (frequency of anxiety) would entail a response of 0 to item 2 (intensity of anxiety) [25].

Construct validity was examined through correlations with measures of anxiety (BAI), depression (BDI-II), positive and negative affect (PANAS-P and PANAS-N), and quality of life (QLI). Cohen’s [48] benchmarks for the interpretation of the correlation values were used, where effect sizes between .10 and .30 are considered small, those between .30 and .50 are considered medium, and those of .50 or above are considered large.

To assess the sensitivity and specificity of the OASIS scores in detecting anxiety symptoms, cutoff scores of the BAI scores were used to classify participants between those without anxiety (BAI score < 10) and with anxiety (BAI score ≥10) [49]. The cutoff point on the BAI to assess the sensitivity and specificity of the OASIS scores was 10, so that BAI scores ≥10 were considered to reflect anxiety symptoms. To examine the precision of the OASIS scores in detecting cases with and without anxiety symptoms, the receiver operating characteristic (ROC) curve was calculated, as well as the area under the curve (AUC). The AUC is a quantitative index that combines sensitivity and specificity in order to provide information about the precision of a test score as a proportion, so that the larger the proportion, the greater the precision of the test. The sensitivity of test scores is the proportion of positive cases (i.e., participants with anxiety, assessed with the BAI) that are correctly identified by the OASIS scores. The specificity of test scores is the proportion of negative cases (i.e., participants without anxiety, assessed by the BAI) correctly identified by the OASIS scores as the best result. AUC values under .5 will reflect lack of precision, whereas AUC values above .9 indicate excellent precision, values between .7 and .9 indicate moderate precision, and values between .5 and .7 indicate mild precision. The AUC represents the probability that a participant randomly selected from the group with anxiety will obtain a higher score on the OASIS than another participant, also randomly selected, from the group of people without anxiety. A 95% confidence interval around the AUC and its statistical significance were also calculated [50]. Sensitivity and specificity were calculated for each cutoff point, as well as Positive Predictive Values (PPV), Negative Predictive Values (NPV), and their corresponding 95% confidence intervals. PPV represents the proportion of cases correctly identified by the OASIS as positive with regard to all the positive cases, whereas NPV represents the proportion of cases correctly identified as negative by the OASIS with regard to all the negative cases. In order to identify the optimum cutoff point for the OASIS, four methods were applied to each cutoff score [51]: the Youden index (*J)*, Index of Union (*IU)*, Closest to (0, 1) Criteria (*ER*), and Concordance Probability Method (*CZ*). The Youden index is defined as *J* = max(*Sensitivity* + *Specificity* -1), so that the OASIS cutoff point that correspond to the maximum *J* value is considered the optimal cutoff point. The Index of Union (*IU*) was calculated as *IU* = min(|*Sensitivity*–*AUC*| + |*Specificity*—*AUC*|). The *IU* is calculated to guarantee that the sensitivity and specificity obtained at this cutoff point is simultaneously close to the *AUC* value, and the difference between the sensitivity and specificity obtained at this cutoff point should be minimal. The Closest to (0, 1) Criteria is calculated as $ER=\sqrt{{(1-Sensitivity}^{2}+{\left(1-Specificity\right)}^{2}}$, and the optimal cutoff point according to this index is defined as the point closest to the point (0, 1) on the ROC curve. Finally, the Concordance Probability Method defines the optimal cutoff point as the point that maximizes the product of sensitivity and specificity. *CZ* is calculated as *CZ* = Sensitivity*Specificity. The OASIS score that met the four criteria, or most of them, was selected as the optimal cutoff point.

Finally, in order to analyze sensitivity of OASIS scores to change, means and standard deviations for the pretest and posttest were calculated with the OASIS scores from two studies about the efficacy of Internet CBT in patients with emotional disorders. Part of the total sample completed the OASIS before and immediately after receiving an Internet-based treatment. Thus, 24 patients received Smiling is Fun [52] (hereinafter *subsample 1*), and 68 patients received Emotion Regulation [53,54] (hereinafter *subsample 2*). Smiling is Fun is an 8-module Internet-based treatment for depression that includes components of evidence-based treatments. The protocol stresses the importance and benefits of being active and staying involved in life, values, and goals. It allows the individual to learn and practice adaptive ways to cope with depressive symptoms and confront daily problems. Specifically, some components of Barlow’s Unified Protocol (UP) have been adapted, namely, motivation, psychoeducation, cognitive therapy, and relapse prevention [55]. Furthermore, the program incorporates a Behavioral Activation component [56] and a Positive Psychology component, which includes strategies to promote and enhance personal strengths, positive feelings, positive cognitions, and positive behavior [57,58]. Emotion Regulation is a 12-module transdiagnostic Internet-based treatment for anxiety and depressive disorders. The treatment protocol is delivered through a multimedia web platform (https://www.psicologiaytecnologia.com/) (with videos, images, printable documents, etc.), which allows participants easy and optimal use on a PC or tablet. The content of the protocol is adapted from the Unified Protocol [59] and from Marsha Linehan’s protocol [60], with four core components: present-focused emotional awareness, cognitive flexibility, behavioral and emotional avoidance patterns, and interoceptive and situational exposure. The protocol also includes traditional therapeutic components of evidence-based treatments, such as psychoeducation, motivation for change, and relapse prevention.

Minimum and maximum OASIS scores were also obtained from the pretest to check potential floor or ceiling effects. Evidence of floor or ceiling effects is present when more than 17% of the participants obtained the lowest or highest possible score on the test, respectively (in our case, 0 and 20). In addition, t-tests were applied to test the statistical significance of the pretest-posttest mean differences. To quantify the OASIS scores’ sensitivity to change, the standardized mean change index was used as the effect size, defined as the difference between the pretest and the posttest means divided by the standard deviation of the change scores. The positive bias of the d index for small sample sizes was corrected with the c(m) correction factor [61]:

$$d=c(m){\bar{y}}_{Pre}-{\bar{y}}_{Post}/{SD}_{Change}$$

with ${\bar{y}}_{Pre}$ and ${\bar{y}}_{Post}$ being the pretest and posttest means, and c(m) being:

c(m)=1−3/4N−5

In addition, 95% confidence intervals for the d indices were calculated by means of d ± 1.96xSE(d), with 1.*96* being the 97.5 percentile of the standard normal distribution, and SE(d) being the standard error of the d index [61]:

$$SE(d)=\sqrt{{c(m)}^{2}x(1/n)x(n-1/n-3)x(1+nd^{2})-d^{2}}$$

All of these calculations were applied separately for subsamples 1 and 2. To offer a contextualized interpretation of the d indices obtained in subsamples 1 and 2, we used the results of a systematic review of meta-analyses carried out on the efficacy of psychological treatments that applied the standardized mean change index as the effect size [62]. In this review, percentiles 25, 50, and 75 of the d indices’ distribution were 0.64, 0.75, and 1.26. Therefore, a reasonable interpretation of these three values is to consider them as reflecting low, moderate, and large magnitudes of the effect.

CFA was calculated using the EQS program, version 6.1. Sensitivity, specificity, PPV, and NPV were calculated using a web application (http://vassarstats.net/clin1.html). The software SPSS Statistics version 22.0 was used for the remaining analyses.

## Results

### Preliminary analyses

The mean OASIS score was 8.69 (SD = 4.21) in the total sample (n = 583), 8.92 (SD = 4.28) for females (n = 421), and 8.15 (SD = 3.96) for the male participants (n = 162). Tables 2 and 3 show descriptive statistics for each item and the total score on the OASIS, and for the remaining instruments, for both the depressive and anxiety disorder samples, respectively.

**Table 2:** Descriptive statistics for each item and the total score on the OASIS in depressive and anxiety disorder samples.

	Anxiety sample (n = 250)				Depression sample (n = 333)			
	M	SD	λ_1_	λ_2_	M	SD	λ_1_	λ_2_
**Item 1**	1.96	1.06	.108	-.466	2.05	1.04	-.145	-.881
**Item 2**	1.80	.88	-.157	-.911	1.79	.86	-.285	-.124
**Item 3**	1.56	1.16	.330	-.049	1.65	1.13	.246	-.684
**Item 4**	1.61	1.08	.229	-.723	1.70	1.05	-.087	-.925
**Item 5**	1.53	1.11	.371	-.808	1.70	1.06	-.045	-.697
**Total score**	8.37	4.29	.230	-.624	8.96	4.17	-.190	-.420

M = Mean; SD = Standard deviation; λ_1_ = skewness; λ_2_ = kurtosis

**Table 3:** Descriptive statistics for convergent and discriminant validity measures in depressive and anxiety disorder samples.

	Anxiety sample				Depression sample			
	M	SD	λ_1_	λ_2_	M	SD	λ_1_	λ_2_
**BAI**	20.41	11.94	.451	-.588	19.13	11.39	-.690	-.008
**BDI-II**	21.74	11.45	.284	-.339	24.70	10.70	.409	.331
**ODSIS**	6.41	4.93	.384	-.899	8.64	4.68	-.107	-.778
**PANAS-P**	23.45	8.13	.695	.073	19.64	6.53	.828	.317
**PANAS-N**	27.24	7.81	.110	-.574	25.32	7.98	.286	-.282
**QLI**	4.94	1.66	.041	-.473	4.51	1.71	.275	-.407

M = Mean; SD = Standard deviation; λ_1_ = skewness; λ_2_ = kurtosis; BAI = Beck Anxiety Inventory; BDI-II = Beck Depression Inventory-II; ODSIS = Overall Depression Severity and Impairment Scale; PANAS-P = Positive and Negative Affect Schedule-Positive Affect; PANAS-N = Positive and Negative Affect Schedule-Negative Affect; QLI = Multidimensional Quality of Life Questionnaire

Significant differences were found in the OASIS scores based on the number of comorbid disorders (F = 6.91; p < .001), with higher anxiety levels found the participants with a larger number of comorbid disorders. There were no significant differences based on sex, civil status, education level, or principal diagnosis. In addition, no statistical relationships were observed between the participants’ age and OASIS scores.

### Factor structure

**Fig 1:**
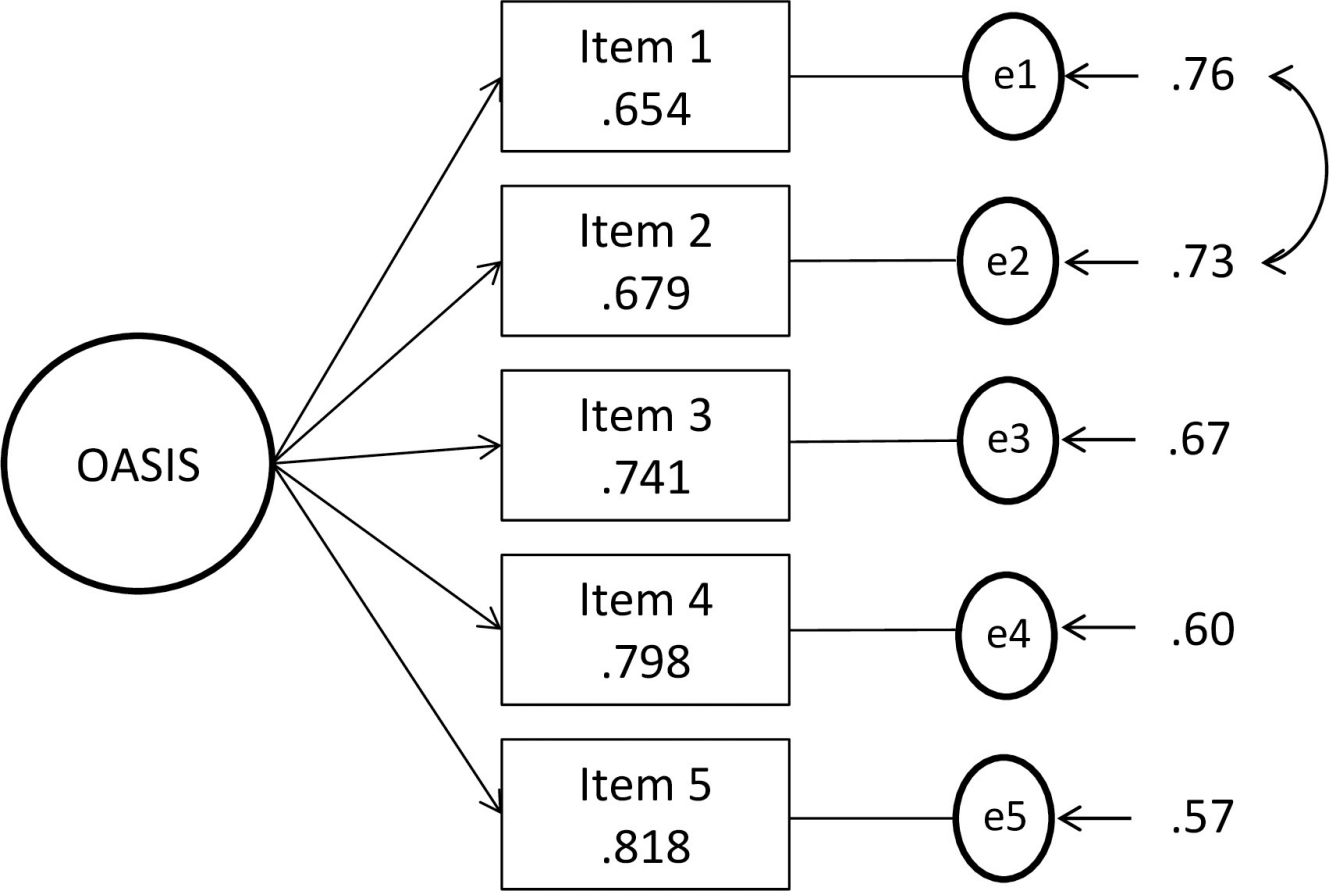
Confirmatory factor analysis (CFA) model. Rectangles are measured variables, the large circle is the latent construct, and small circles are residual variances. Factor loadings are standardized. All values are significant at *p* < .05. The solution specified correlated error variance between items 1 and 2.

A single-factor model resulted in an adequate model fit: χ^2^_4_ = 11.693, p > .01; SRMR = .027; RMSEA = .058, 90% CI [.015, .104]; CFI = .995. Factor loadings showed that all the items were strongly related to this factor, with values ranging from .65 to .82 All these values reached significance at *p* < .05 (see Fig 1).

### Internal consistency

Cronbach’s alpha for the five items on the OASIS was .86. Table 4 shows the results for Cronbach’s alpha when omitting items, corrected correlations between each item and the total score, and correlations between the five items of the OASIS. The results obtained indicate good internal consistency of the OASIS that would not be increased by excluding any item.

**Table 4:** Cronbach’s alpha if item is deleted, corrected item-total score correlation, and correlations between items.

	Cronbach’s Alpha if item deleted	Corrected item-total correlation	Correlations between items				
			Item 1	Item 2	Item 3	Item 4	Item 5
**Item 1**	.842	.658	1				
**Item 2**	.838	.692	.689*	1			
**Item 3**	.849	.640	.422*	.464*	1		
**Item 4**	.825	.726	.560*	.585*	.587*	1	
**Item 5**	.824	.729	.543*	.543*	.643*	.628*	1

*Correlation was significant at *p* < .01 (two-tailed)

### Convergent validity

Table 5 shows the correlation between the OASIS and the convergent validity measures. A large positive correlation was expected between the OASIS and the BAI. A positive but medium correlation was expected between the OASIS and the BDI-II. Given the theoretical and empirical associations between the dimensions of positive and negative affect and anxiety [2], we anticipated a positive but medium correlation between the OASIS and the PANAS-N, and a negative and medium correlation with the PANAS-P. Finally, we anticipated a negative and medium correlation between the OASIS and QLI (quality of life). All these results were interpreted as evidence for convergent validity.

**Table 5:** Correlations of the OASIS with convergent validity measures.

	OASIS	BAI	ODSIS	BDI-II	PANAS-P	PANAS-N	QLI
**OASIS**	-	.61*	.65*	.60*	-.40*	.60*	-.58*
**BAI**		-	.35*	.47*	-.25*	.53*	-.41*
**ODSIS**			-	.67*	-.57*	.49*	-.69*
**BDI-II**				-	-.56*	.57*	-.76*
**PANAS-P**					-	-.32*	.71*
**PANAS-N**						-	-.48*
**QLI**							-

*Correlation was statistically significant at *p* < .01 (two-tailed)

OASIS = Overall Anxiety Severity and Impairment Scale, BAI = Beck Anxiety Inventory, ODSIS = Overall Depression Severity and Impairment Scale, BDI-II = Beck Depression Inventory-II, PANAS-P = Positive and Negative Affect Schedule-Positive Affect, PANAS-N = Positive and Negative Affect Schedule-Negative Affect, QLI = Multidimensional Quality of Life Questionnaire

The OASIS correlated significantly with all the measures. As predicted, positive and large correlations were found between the OASIS and the BAI (*r* = .61, *p* < .01). In addition, large and positive correlations were found between the OASIS and the BDI-II (*r* = .60, *p* < .01), and between the OASIS and the ODSIS (*r* = .65, *p* < .01). The OASIS correlated largely with the PANAS-N (*r* = .60, *p* < .01). Finally, the analyses yielded a negative medium correlation between the OASIS and the PANAS-P (*r* = -.40, *p* < .01), and a negative large correlation between the OASIS and the QLI (r = -.58, p < .01).

### Receiver operating characteristic (ROC)

A ROC curve was calculated in the sample when a cutoff point ≥ 10 was applied to the BAI scores. The AUC obtained was .817 (95%CI: .731 and .903) and reached statistical significance (*p* < .001). This AUC can be interpreted as indicating that there was a .817 probability of randomly selecting a participant from the anxiety group (i.e., with a BAI score ≥ 10) with an OASIS score higher than that of any other participant, also randomly selected, from the group without anxiety (i.e., with BAI score < 10). An AUC = .817 can also be interpreted as reflecting moderate precision from a clinical point of view. Therefore, the precision of the OASIS scores in detecting any type of anxiety (mild, moderate, or severe) can be considered to have a moderate magnitude. Table 6 presents the sensitivity, specificity, PPV, and NPV obtained with the OASIS scores for the cutoff point ≥ 10 on the BAI. The table also shows the results of the four methods used to select the optimal cutoff score for the OASIS (Youden index, *J*, Index of Union, *IU*, the Closest to (0, 1) Criteria, *ER*, and the Concordance Probability Method, *CZ*). The OASIS score = 7.5 met three of the four criteria (*IU*, *ER*, and *CZ* criteria); regarding the Youden index, this score obtained the second best value (.498), very close to the maximum value obtained with this method (.503). Therefore, 7.5 was selected as the optimal cutoff point to detect anxiety symptoms (i.e., OASIS scores over 7 indicate anxiety symptoms). For this cutoff point, sensitivity was .727 (95%CI: .650; .792), and specificity was .771 (95% CI: .594; .889). PPV was .936 (95% CI: .874; .970), and NPV was .380 (95% CI: .270; .504).

**Table 6:** Statistics to assess the diagnostic accuracy of the OASIS scores.

OASIS score	*Se*	*Sp*	*PPV*	*NPV*	*J*	*IU*	*ER*	*CZ*
0.5	.994	.171	.846	.857	.165	.823	.829	.170
1.5	.994	.200	.851	.875	.194	.794	.800	.199
2.5	.975	.314	.867	.733	.289	.661	.686	.306
3.5	.969	.457	.891	.762	.426	.512	.544	.443
4.5	.932	.571	.909	.645	**.503**	.361	.434	.532
5.5	.876	.600	.910	.512	.476	.276	.419	.526
6.5	.814	.657	.916	.434	.471	.163	.390	.535
**7.5**	**.727**	**.771**	**.936**	**.380**	**.498**	**.136**	**.356**	**.561**
8.5	.627	.800	.935	.318	.427	.207	.423	.502
9.5	.528	.857	.944	.283	.385	.329	.493	.452
10.5	.435	.914	.959	.260	.349	.479	.572	.398
11.5	.354	.914	.950	.235	.268	.560	.652	.324
12.5	.242	.914	.928	.208	.156	.672	.763	.221
13.5	.161	.971	.963	.201	.132	.810	.840	.156
14.5	.124	1	1	.199	.124	.876	.876	.124
15.5	.093	1	1	.193	.093	.907	.907	.093
16.5	.043	1	1	.185	.043	.957	.957	.043
17.5	.019	1	1	.181	.019	.981	.981	.019
18.5	.006	1	1	.179	.006	.994	.994	.006
20	0	1	NA	NA	0	1	1	0

*Se* = Sensitivity; *Sp* = Specificity; *PPV* = Positive Predictive Value; *NPV* = Negative Predictive Value; *J* = Youden index; *IU* = Index of Union; *ER* = Closest to (0, 1) Criteria; *CZ* = Concordance Probability Method; NA = Not applicable.

### Analysis of sensitivity to change

Two subsamples were used for the analysis of sensitivity to change. Subsample 1 consisted of 24 patients who completed an Internet-based treatment for depression [52], and subsample 2 was made up of 68 patients who underwent a transdiagnostic Internet-based treatment for anxiety and depressive disorders [53,54]. To examine potential floor and ceiling effects for the OASIS scores, the frequency and percentage of minimum (0) and maximum (20) scores was tabulated for subsamples 1 and 2 on the pretest. The results showed that only 2 patients out of 24 (12%) in subsample 1, and 3 out of 68 in subsample 2 obtained a score of 0 (minimum). In addition, no patient in any of the subsamples obtained a score of 20 (maximum). Therefore, evidence of floor and ceiling effects can be ruled out, as the percentage was lower than 17% in all cases.

To examine the sensitivity to change of the OASIS scores, means and standard deviations were calculated for each subsample, both on the pretest and the posttest. The statistical significance of the pretest-posttest change scores was assessed by applying t-tests, which, as Table 7 reveals, were statistically significant for both studies. The clinical significance was assessed by means of the effect size index ‘standardized mean change index’ (d). Following Rubio-Aparicio et al. results [62], subsamples 1 and 2 obtained *d* indices that can be interpreted as reflecting moderate (*d* = 0.72) and moderate-to-large (d = 0.90) clinical relevance, respectively.

**Table 7:** Descriptive and inferential results from the two subsamples for the OASIS scores on the pretest and the posttest.

Subsample	N	Pretest		Posttest		*t*	*d*
		Mean	SD	Mean	SD		
**1**	24	6.42	3.46	3.00	2.62	3.65***	0.72 [0.26, 1.18]
**2**	68	8.59	4.41	4.50	4.60	7.44***	0.90 [0.61, 1.19]

***p < .001

N = sample size; SD = standard deviation; *t* = *t* statistic for testing the pretest-posttest mean difference; *d* = standardized mean change index (95% CI in brackets).

## Discussion

The aim of this study was to analyze the psychometric properties of the online version of the OASIS in a Spanish sample of patients with emotional disorders. This study evaluated the reliability, construct validity, and latent structure of the OASIS. In addition, cutoff scores were obtained, and sensitivity to change was examined.

First, preliminary analyses showed that patients with more comorbid disorders were significantly more anxious than patients with fewer comorbid disorders, a finding that was somewhat expected given the strong association observed between comorbidity and severity [63]. By contrast, no statistically significant differences were found based on sex, education level, marital status, or principal diagnosis (i.e. anxiety disorder vs. depressive disorder), which, taken together, suggests that the Spanish version of the OASIS can be used indistinctly across patients with different sociodemographic and clinical characteristics. In this vein, it is important to note that a large proportion of patients in this study (53.5%) presented at least with one anxiety or depressive disorder. Second, regarding reliability, the five items on the OASIS demonstrated good internal consistency (alpha = .86). Third, as in previous validations of the instrument [25–27], confirmatory factor analysis revealed a unidimensional factor structure. Moreover, as expected, the model showed correlated error variance between items 1 and 2.

Regarding the ROC analysis, a cutoff point of 7.5 was found to meet three of the four criteria used to select the optimal cutoff point (i.e. Index of Union, Closest to (0, 1) Criteria, and Concordance Probability Method). These findings suggest that this score (i.e. scores above 7 at the OASIS) can be used as a cutoff point to discriminate between patients with anxiety symptoms of clinical consideration vs. anxiety symptoms of no clinical consideration. This information might be useful, for instance, for screening and selecting patients with anxiety symptoms for clinical trials. The results obtained in this study using ROC curves are consistent with prior validations of the instrument in clinical populations, which showed that cutoff scores of around 8 differentiate anxious patients from non-anxious patients [25,27].

This study also examined sensitivity to change by analyzing the significance of the improvements from pre- to post-treatment on the OASIS scores. The analyses showed moderate to large effect sizes (Cohen’s d between .72 and .90), which suggests that the scale can not only be used for screening purposes (i.e. by using the cut-off point), but also that it is able to detect changes in anxiety and therefore it can be used to examine the impact of an intervention.

Regarding construct validity, positive and large correlations were found between the OASIS and the BAI, as anticipated, which is interpreted in this study as evidence of adequate convergent validity with one of the most widely used questionnaires for the assessment of anxiety. The fact that the OASIS also correlated significantly with measures of positive and negative affectivity, but less than with measures of anxiety (i.e. BAI), was interpreted as evidence for the discriminant validity of the instrument. Finally, although we predicted medium correlations between the OASIS and the depression measures (i.e. ODSIS and BDI-II), the results showed large correlations between these measures. In this regard, it is important to note that a large proportion of the patients (53.5%) had comorbid depressive or anxiety disorders, which might account for the large correlations between anxiety and depression obtained in this study. Overall, the results obtained in this study were interpreted as evidence for construct validity.

Overall, the results of this study are consistent with those obtained in prior validations of the scale [25,31,32], and they support the adequacy of the OASIS as a valid measure for the online assessment of the anxiety severity and impairment associated with anxiety symptoms.

This study has several strengths. First, to the best of our knowledge, this is the first study to evaluate the psychometric properties of the OASIS in a Spanish clinical sample of individuals with anxiety and depressive disorders. Brief instruments to assess the severity and impairment related to anxiety are lacking in Spain, and so this study contributes to filling the gap in this particular field. Second, although the OASIS has already been validated in transdiagnostic samples with emotional disorders [31], the sample size of patients with principal diagnoses of a depressive disorder was larger in this study. Unlike the study by Bragdon et al. [31], in which most patients had a principal diagnosis of an anxiety disorder (85.6%), we used a sample with a more balanced number of patients with a principal diagnosis of anxiety (55.2%) versus depression (44.8%). Given the burden and prevalence of depression, as well as its transdiagnostic nature and high comorbidity rates with anxiety disorders [2], the findings obtained in this study contribute to the literature on the OASIS by providing data about how the scale performs in patients with a principal diagnosis of depression. Third, the large sample size used in this study (n = 583), and its high diagnostic heterogeneity (i.e. individuals with a variety of anxiety and depressive disorders), helps to increase the generalizability of the results obtained in the study. Fourth, although various validations of the OASIS have been performed in clinical samples [25,31,33,64], none of them have analyzed how the scale performs as a treatment outcome measure. Following previous recommendations [31,64], in this study we intended to contribute to filling this gap by analyzing sensitivity to change in two subsamples of patients who received Internet treatments. Fifth, all the patients in the study completed the OASIS through online surveys. Therefore, the results obtained in this study suggest that the online version of the OASIS is an adequate instrument for the online assessment of anxiety (e.g. in trials examining Internet treatments, where both the assessment and the treatment are delivered through an online platform). Given the proliferation of Internet-based treatments in the past decade, the need for validated online assessment instruments is greater than ever before.

### Limitations

This study has some limitations that should be acknowledged. First, test-retest reliability was not evaluated in this study. Because all the participants in this study were derived from clinical samples that were receiving treatment, we were not able to analyze this aspect. Second, we were not able to analyze sensitivity to change with the entire sample because scores from pre- to post-treatment were not available for all participants in this study. Moreover, we were not able to examine the sensitivity to change of the OASIS compared to other scales for the assessment of anxiety, such as the BAI. Third, it might have been useful to include additional measures of anxiety in this study to further evaluate the convergent validity of the OASIS. However, it is important to note that the inclusion of instruments in this study was determined by the fact that all the patients were derived from trials where the selection of instruments was already pre-specified. For this reason, only two measures for the assessment of anxiety were used in this study (OASIS and BAI). Fourth, even though the BAI is a well-established measure and one of the more widely used scales for the assessment of anxiety [65–67], we did not follow the optimum approach for the calculation of the ROC curve because the classification of subjects was based on a cutoff from a scale (BAI) rather than a group of healthy control individuals. Hence, the cutoff score obtained in this study should be considered with caution. Finally, the proportion of females and males in this study was not balanced, which might affect the representativity of the results. However, the proportion of females versus males in this study is likely to have been affected by the higher prevalence rates of anxiety and depressive disorders in females compared to males [68].

## Conclusions

In conclusion, the results obtained in this study support the adequacy of the online version of the OASIS in clinical samples of Spanish patients with anxiety and depressive disorders. The brevity and ease of use of the OASIS makes this scale an adequate tool for the quick screening of the severity and impairment associated with anxiety. Future validations of the OASIS should analyze its sensitivity to change in comparison with other measures of anxiety, in order to draw firmer conclusions about this aspect.

The psychometric properties of the online version of the OASIS were analyzed in this study. Similarly to evidence-based online treatments, the validation of online scales can have a direct impact in the dissemination of evidence-based methods for the assessment of behavioral, cognitive and psychopathological processes.

## Supporting information

S1 Appendix OASIS (Spanish version). (DOCX)

S2 Appendix OASIS (English version). (DOCX)
