# Supplementary material for: A brief online transdiagnostic measure: Psychometric properties of the Overall Anxiety Severity and Impairment Scale (OASIS) among Spanish patients with emotional disorders
Source: PLoS One. 2018 Nov 1;13(11):e0206516. doi: 10.1371/journal.pone.0206516 (PMC6211825; doi:10.1371/journal.pone.0206516)
Supplement: S1 Appendix — (DOCX) [file pone.0206516.s001.docx]

**Overall Anxiety Severity and Impairment Scale (OASIS)**

Los siguientes ítems preguntan sobre ansiedad y miedo. Para cada ítem, selecciona el número que mejor describe tu experiencia durante la última semana.

**1. Durante la última semana, ¿con qué frecuencia te has sentido ansioso?**

0 = No me sentí ansioso durante la última semana.

1 = Ansiedad infrecuente. Me sentí ansioso en algunos momentos.

2 = Ansiedad ocasional. La mitad del tiempo me sentí ansioso y la otra mitad no. Me costó relajarme.

3 = Ansiedad frecuente. Me sentí ansioso la mayor parte del tiempo. Me resultó muy difícil relajarme.

4 = Ansiedad constante. Me sentí ansioso todo el tiempo y nunca llegué a relajarme.

**2. Durante la última semana, cuando te sentiste ansioso, ¿en qué medida tu ansiedad fue intensa o severa?**

0 = Poco o nada. La ansiedad estuvo ausente o casi no la noté.

1 = Leve. La ansiedad fue de baja intensidad. Pude relajarme cuando lo intenté. Los síntomas físicos fueron sólo un poco molestos.

2 = Moderada. La ansiedad me generó malestar en algunos momentos. Me resultó difícil relajarme o concentrarme, pero pude hacerlo cuando lo intenté. Los síntomas físicos fueron molestos.

3 = Severa. La ansiedad fue intensa la mayor parte del tiempo. Me resultó muy difícil relajarme o concentrarme en cualquier otra cosa. Los síntomas físicos fueron enormemente molestos.

4 = Extrema. La ansiedad me sobrepasó. Me fue totalmente imposible relajarme. Los síntomas físicos fueron insoportables.

**3. Durante la última semana, ¿con qué frecuencia evitaste situaciones, lugares, objetos o actividades debido a tu ansiedad o miedo?**

0 = Ninguna. No evité lugares, situaciones, actividades o cosas por miedo.

1 = Infrecuente. Evité algunas cosas de vez en cuando, pero por lo general me enfrenté a las situaciones u objetos. Mi estilo de vida no se vio afectado.

2 = Ocasional. Tuve algo de miedo a ciertas situaciones, lugares u objetos, pero todavía pudo manejarlos. Mi estilo de vida sufrió pocos cambios. Siempre o casi siempre evité las cosas que me dan miedo si estaba solo, pero las pude manejar si alguien venía conmigo.

3 = Frecuente. Tuve bastante miedo y realmente intenté evitar las cosas que me asustan. He hecho cambios significativos en mi estilo de vida para evitar objetos, situaciones, actividades o lugares.

4 = Todo el tiempo. Evitar objetos, situaciones, actividades o lugares ha ocupado gran parte de mi vida. Mi estilo de vida se ha visto enormemente afectado y ya no hago cosas con las que solía disfrutar.

**4. Durante la última semana, ¿en qué medida ha interferido la ansiedad en tu capacidad para hacer las cosas que tenías que hacer respecto al trabajo, el colegio o tu hogar?**

0 = Nada. La ansiedad no interfirió en mi trabajo/hogar/colegio.

1 = Leve. La ansiedad me causó algo de interferencia en mi trabajo/hogar/colegio. Las cosas eran más difíciles, pero pude realizar todo lo que necesitaba hacer.

2 = Moderada. La ansiedad definitivamente interfirió en mis tareas. He podido realizar la mayoría de las cosas, pero sólo algunas las he hecho tan bien como en el pasado.

3 = Severa. La ansiedad verdaderamente ha cambiado mi capacidad para hacer las cosas. Algunas cosas las he podido realizar, pero otras no. Mi rendimiento se ha visto definitivamente afectado.

4 = Extrema. La ansiedad ha llegado a ser incapacitante. He sido incapaz de completar mis tareas y he tenido que irme del colegio, he dejado o me han despedido de mi trabajo o he sido incapaz de completar las tareas del hogar y he sufrido consecuencias como desalojos, cobradores, etc.

**5. Durante la última semana, ¿en qué medida ha interferido la ansiedad en tu vida social y en tus relaciones?**

0 = Nada. La ansiedad no interfirió en mis relaciones.

1 = Leve. La ansiedad apenas interfirió en mis relaciones. Algunas de mis amistades y otras relaciones se han visto afectadas, pero en conjunto mi vida social sigue siendo satisfactoria.

2 = Moderada. La ansiedad interfirió algo en mi vida social, pero sigo teniendo algunas relaciones cercanas. No paso tanto tiempo con otros como en el pasado, pero sigo teniendo relaciones sociales algunas veces.

3 = Severa. Mis amistades y otras relaciones se han visto muy afectadas a causa de mi ansiedad. No disfruto de las actividades sociales. Tengo muy pocas relaciones sociales.

4 = Extrema. La ansiedad ha alterado completamente mis actividades sociales. Todas mis relaciones se han visto afectadas o han finalizado. Mi vida familiar es extremadamente tensa.
