# Supplementary material for: A brief online transdiagnostic measure: Psychometric properties of the Overall Anxiety Severity and Impairment Scale (OASIS) among Spanish patients with emotional disorders
Source: PLoS One. 2018 Nov 1;13(11):e0206516. doi: 10.1371/journal.pone.0206516 (PMC6211825; doi:10.1371/journal.pone.0206516)
Supplement: S2 Appendix — (DOCX) [file pone.0206516.s002.docx]

**Overall Anxiety Severity and Impairment Scale (OASIS)**

The following items ask about anxiety and fear. For each item, select the number for the answer that best describes your experience over the past week.

**1. In the past week, how often have you felt anxious?**

0 = No anxiety in the past week.

1 = Infrequent anxiety. Felt anxious a few times.

2 = Occasional anxiety. Felt anxious as much of the time as not. It was hard to relax.

3 = Frequent anxiety. Felt anxious most of the time. It was very difficult to relax.

4 = Constant anxiety. Felt anxious all of the time and never really relaxed.

**2. In the past week, when you have felt anxious, how intense or severe was your anxiety?**

0 = Little or None: Anxiety was absent or barely noticeable.

1 = Mild: Anxiety was at a low level. It was possible to relax when I tried. Physical symptoms were only slightly uncomfortable.

2 = Moderate: Anxiety was distressing at times. It was hard to relax or concentrate, but I could do it if I tried. Physical symptoms were uncomfortable.

3 = Severe: Anxiety was intense much of the time. It was very difficult to relax or focus on anything else. Physical symptoms were extremely uncomfortable.

4 = Extreme: Anxiety was overwhelming. It was impossible to relax at all. Physical symptoms were unbearable.

**3. In the past week, how often did you avoid situations, places, objects, or activities because of anxiety or fear?**

0 = None: I do not avoid places, situations, activities, or things because of fear.

1 = Infrequent: I avoid something once in a while, but will usually face the situation or confront the object. My lifestyle is not affected.

2 = Occasional: I have some fear of certain situations, places, or objects, but it is still manageable. My lifestyle has only changed in minor ways. I always or almost always avoid the things I fear when I’m alone, but can handle them if someone comes with me.

3 = Frequent: I have considerable fear and really try to avoid the things that frighten me. I have made signifi cant changes in my lifestyle to avoid the object, situation, activity, or place.

4 = All the Time: Avoiding objects, situations, activities, or places has taken over my life. My lifestyle has been extensively affected and I no longer do things that I used to enjoy.

**4. In the past week, how much did your anxiety interfere with your ability to do the things you needed to do at work, at school, or at home?**

0 = None: No interference at work/home/school from anxiety.

1 = Mild: My anxiety has caused some interference at work/home/school. Things are more difficult, but everything that needs to be done is still getting done.

2 = Moderate: My anxiety definitely interferes with tasks. Most things are still getting done, but few things are being done as well as in the past.

3 = Severe: My anxiety has really changed my ability to get things done. Some tasks are still being done, but many things are not. My performance has definitely suffered.

4 = Extreme: My anxiety has become incapacitating. I am unable to complete tasks and have had to leave school, have quit or been fired from my job, or have been unable to complete tasks at home and have faced consequences like bill collectors, eviction, etc.

**5. In the past week, how much has anxiety interfered with your social life and relationships?**

0 = None: My anxiety doesn’t affect my relationships.

1 = Mild: My anxiety slightly interferes with my relationships. Some of my friendships and other relationships have suffered, but, overall, my social life is still fulfilling.

2 = Moderate: I have experienced some interference with my social life, but I still have a few close relationships. I don’t spend as much time with others as in the past, but I still socialize sometimes.

3 = Severe: My friendships and other relationships have suffered a lot because of anxiety. I do not enjoy social activities. I socialize very little.

4 = Extreme: My anxiety has completely disrupted my social activities. All of my relationships have suffered or ended. My family life is extremely strained.
